# Supplementary figures and images for: Selective usage of ANP32 proteins by influenza B virus polymerase: Implications in determination of host range
Source: PLoS Pathog. 2020 Oct 12;16(10):e1008989. doi: 10.1371/journal.ppat.1008989 (PMC7580981; doi:10.1371/journal.ppat.1008989)

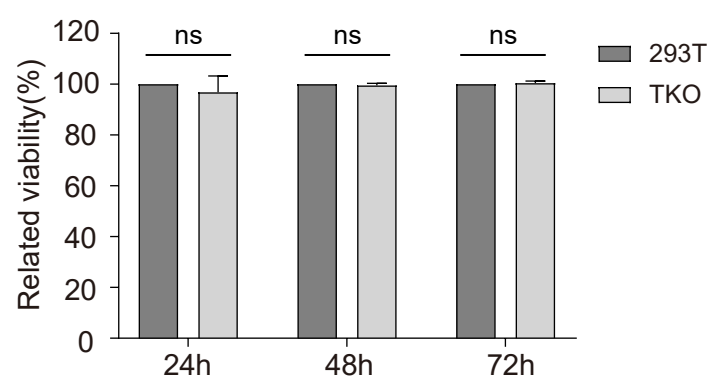

Supplement: S1 Fig — The cell viability of 293T and TKO cells were measured at 24, 48, and 72 h by the CCK-8 reagent in accordance with the manufacturer’s instructions (Beyotime Biotechnology, Shanghai, China). (PDF) [file ppat.1008989.s001.pdf]

B/Yamagata/PJ/2018

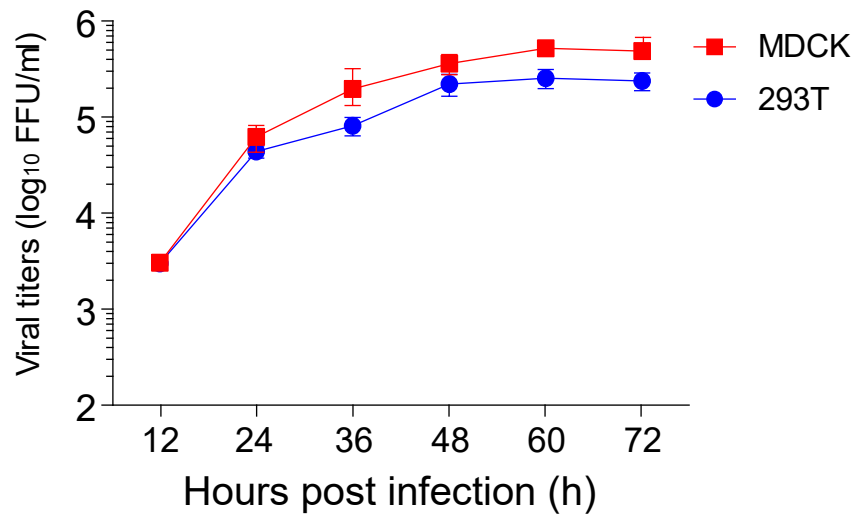

Supplement: S2 Fig — MDCK and 293T cells were infected with B/Yamagata/PJ/2018 virus at a MOI of 0.1. The supernatants were sampled at 12, 24, 36, 48, 60, and 72 h post infection and the viral titers were determined using Fluorescence Focus Units (FFU) assay on MDCK cells. The result is shown as average of n = 3 ± SD. (PDF) [file ppat.1008989.s002.pdf]

A

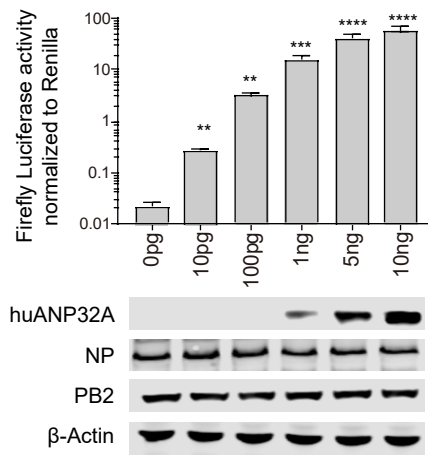

B

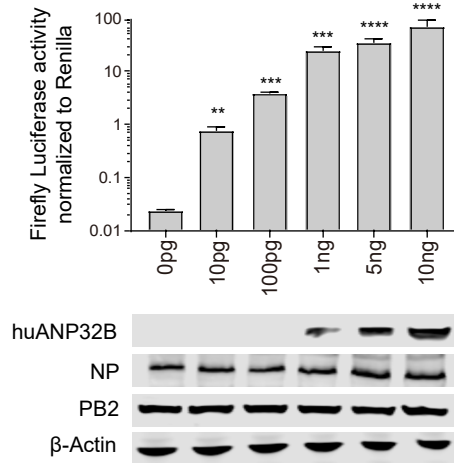

C

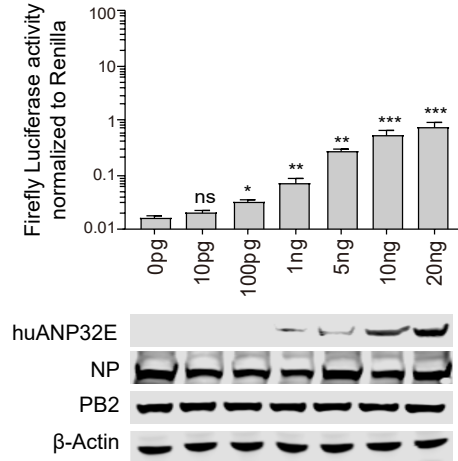

Supplement: S3 Fig — Increasing doses of huANP32A(A), huANP32B(B) or huANP32E(C) were co-transfected with minigenome reporter, Renilla expression control, influenza B virus polymerase of B/Yamagata/1/73 in TKO cells. The expression of ANP32 proteins and polymerase was assessed by western blotting. Luciferase activity was measured 24 h later. (Data are firefly activity normalized to Renilla, Statistical difference between cells were labeled, according to a one-way ANOVA followed by a Dunnett’s test; NS = not significant, *P < 0.05, **P < 0.01, ***P < 0.001, ****P < 0.0001. The results represent at least three independent experiments.) (PDF) [file ppat.1008989.s003.pdf]
