# Supplementary material for: Selective usage of ANP32 proteins by influenza B virus polymerase: Implications in determination of host range
Source: PLoS Pathog. 2020 Oct 12;16(10):e1008989. doi: 10.1371/journal.ppat.1008989 (PMC7580981; doi:10.1371/journal.ppat.1008989)
Supplement: S4 Fig — The protein sequences of ANP32A for human (huANP32A), pig (pgANP32A), equine (eqANP32A), dog (dgANP32A), ostrich(osANP32A), zebra finch (zbANP32A), duck (dkANP32A), turkey (tyANP32A), and chicken (chANP32A) were aligned using the Geneious R10 software. huANP32A was set as the reference sequence. The colors represent similarity of amino acid identity (Black = 100%, dark grey = 80–100%, light grey = 60–80%, white = <60%). Gaps are represented by dashes. Residue numbers correspond to huANP32A. (PDF) [file ppat.1008989.s004.pdf]

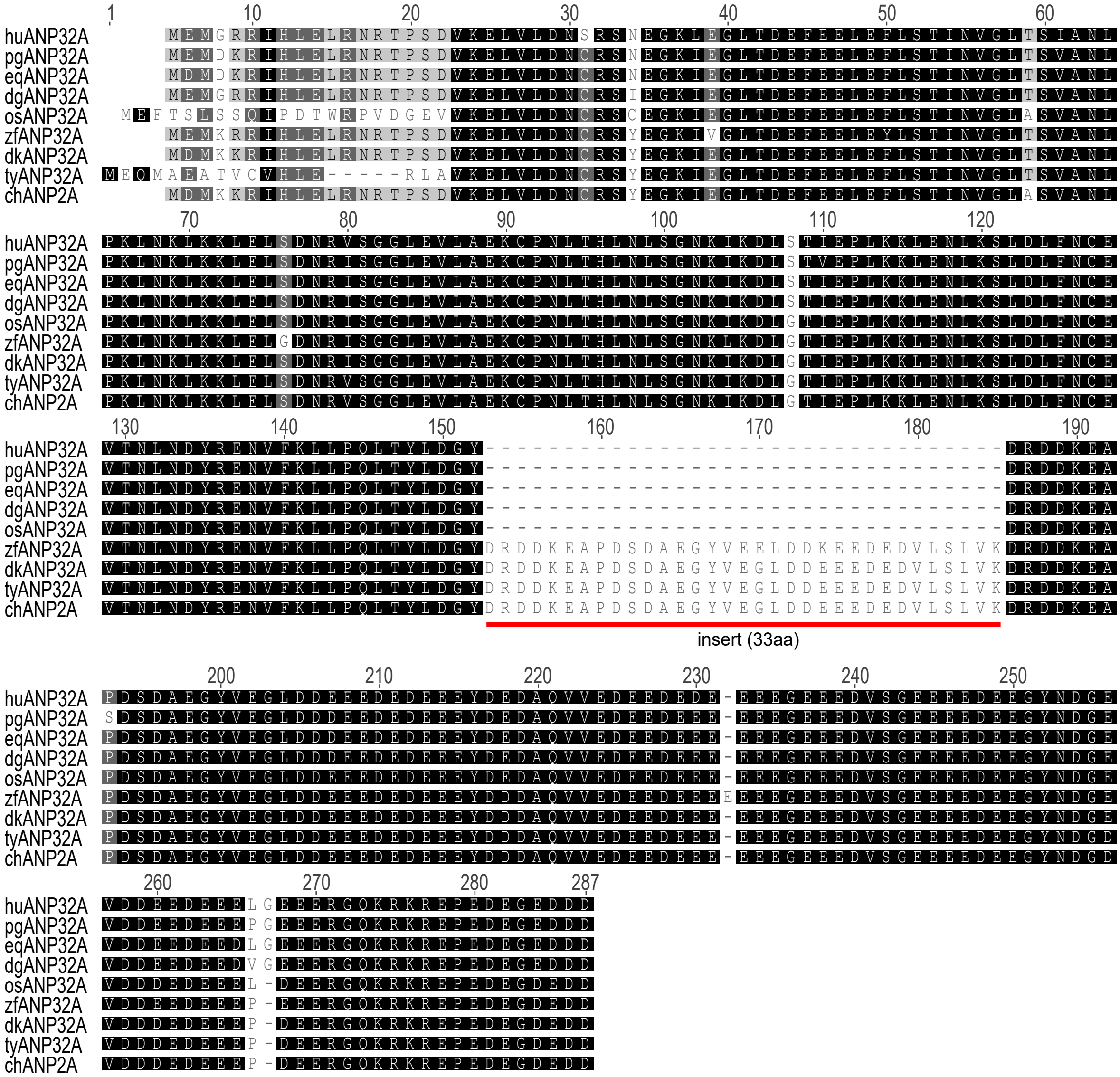
huANP32A
pgANP32A
eqANP32A
dgANP32A
osANP32A
zfANP32A
dkANP32A
tyANP32A
chANP2A
insert (33aa)
