# Supplementary material for: Selective usage of ANP32 proteins by influenza B virus polymerase: Implications in determination of host range
Source: PLoS Pathog. 2020 Oct 12;16(10):e1008989. doi: 10.1371/journal.ppat.1008989 (PMC7580981; doi:10.1371/journal.ppat.1008989)
Supplement: S5 Fig — (A) ANP32 proteins were fusion expressed at downstream of GST-HRV3C peptide in a pCAGGS vector and purified using Glutathione Sepharose 4B and then digested by PreScission Protease. Purified ANP32 proteins were diluted to 100ug/ml and 1ug of the purified protein was checked using SDS-PAGE analysis and western blotting. (B) IBV polymerase PB1, PB2 and PA-His were expressed in 293T cells and purified with Ni Sepharose (GE). The purified protein was checked using SDS-PAGE analysis. (C) The proteins of the purified band in (B) were identified using the mass spectrometry. (PDF) [file ppat.1008989.s005.pdf]

A

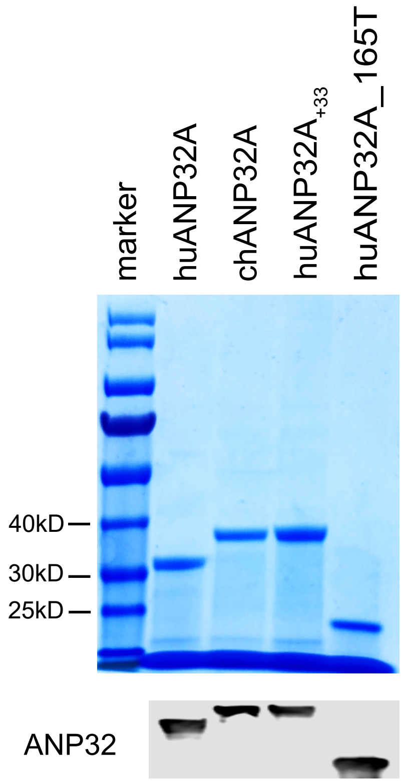

B

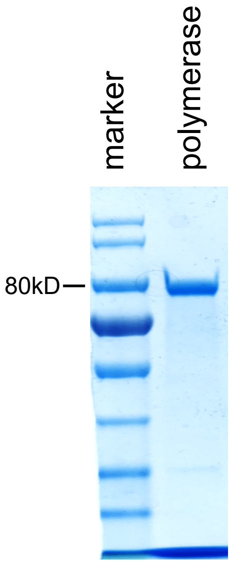

C

Accession	Sum PEP Score	Coverage [%]	# Peptides	# PSMs	# Unique Peptides	# AAs	MW [kDa]	calc. pI	Score Sequest HT: Sequest HT	# Peptides (by Search Engine): Sequest HT
IBV-PA	2097.644	87	156	1448	156	726	83.1	5.76	3907.8	156
IBV-PB2	1966.793	98	174	1341	174	770	87.9	9.17	3486.29	174
IBV-PB1	1315.823	76	115	892	115	752	84.3	8.53	2238.23	115
