# Supplementary material for: Selective usage of ANP32 proteins by influenza B virus polymerase: Implications in determination of host range
Source: PLoS Pathog. 2020 Oct 12;16(10):e1008989. doi: 10.1371/journal.ppat.1008989 (PMC7580981; doi:10.1371/journal.ppat.1008989)
Supplement: Attachment — Submitted filename: Response to reviewers.docx [file ppat.1008989.s006.docx]

Responses to reviewers

<b>Part I - Summary</b></br>Please use this section to discuss strengths/weaknesses of study, novelty/significance, general execution and scholarship.<br/><br/>

Reviewer #1: The authors dissect species-specific functional interactions between ANP32 proteins and influenza viruses, with a focus on influenza B virus. They find that avian ANP32 molecules are generally unable to efficiently support influenza B virus polymerase activity, which is the opposite to avian influenza A virus. Using mutagenesis, binding assays and KO technologies, they map the specific determinants in different ANP32 molecules that are responsible for this effect. The work is notable for its use of constructs from many different species, and its fine dissection of different ANP32 genes (A/B/E) and isoforms. The finding that ANP32E plays a role in influenza B virus replication, but not ANP32A is a highlight. The data are of very high quality and clearly support the conclusion that avian cells may not be permissive for influenza B virus replication due to incompatibility with ANP32s. Taken together with the work on influenza A viruses, this new work is therefore very important as it suggests interesting species-specific evolution of influenza viruses with host ANP32 molecules.

Reviewer #2: Influenza A viruses (IAV) circulate in birds and infect many mammalian species. Although influenza A and B viruses are closely related, IBVs do not infect birds, and the cause of this host range restriction is unknown. ANP32A was shown to be the major species barrier preventing avian IAV polymerases from functioning in mammalian cells. Species-specific differences in ANP32A, ANP32B and ANP32E have since been shown to impact the function of the viral polymerase in different hosts. Here, Zhang, Zhang, et al. thoroughly evaluate the role of ANP32s in supporting influenza B virus polymerase activity. Using a clean genetic background (ANP32A, ANP32B, ANP32E triple knockout; TKO), the authors show that human ANP32A and B support IBV polymerase and that either protein is sufficient for viral replication. ANP32E does not support IAV polymerase, but the authors make the interesting observation that ANP32E provides low levels of activity for the IBV polymerase. None of the avian ANP32 proteins support IBV. Mutant and chimeras reveal that an insertion of acidic residues in human ANP32E contributes to its ability to support IBV polymerase. However, this activity is restricted by the glutamate encoded at amino acid 129; converting this to the asparagine found in ANP32A confers a high degree of activity to both human and avian ANP32E. Thus, ANP32E functions poorly due to variation at aa129, and the species-specific absence of an acidic stretch further eliminates activity in birds, suggesting ANP32E is a major factor restricting IBV host range. Physical interactions between IBV and ANP32A and B are demonstrated by coIP and SPR. The functional assays and dissection of species-specific activities are well-controlled and compelling. However, the authors over-interpret their SPR data, especially in light of how ANP32A interactions do not correlate with support of polymerase activity. Nonetheless, this submission offers strong evidence to explain why influenza B viruses do not circulate in infect birds.

Response:

We thank the reviewer for the comments. We have reworded the sentences on the SPR results (Line 343-347).

Reviewer #3: Influenza B virus is the virus type that together with influenza A viruses (IAV) causes seasonal outbreaks of influenza. A distinctive feature of influenza B virus is the restriction to humans as the main host species, whereas only IAV has established phylogenetic stable lineages in other host species including birds and pigs. The molecular basis for the species specificity of influenza viruses is currently in the focus of intense research and pre-pandemic risc assessment. Previous work has identified the functional interaction of host proteins of the ANP32 family with the viral polymerase complex as a major determinant of host range. While human ANP32-A and -B proteins are essential co-factors of human-adapted polymerases, e.g. chicken ANP32A supports only avian polymerase activity. Several recent papers have unravelled these interactions, the contribution of different ANP32 proteins and the detailed molecular requirements on both viral and host proteins. In this manuscript, Zhang et al describe the requirement of influenza B virus for human ANP32A or B proteins to support polymerase activity and replication in human cells, while chicken orthologues fail to fully support these human-specific viruses. The authors then expand these analyses on a set of ANP32 proteins from other species and demonstrate that the molecular basis for this species specific interaction resides in a 33 amino acid stretch that is exclusive to chicken ANP32A. Further, they investigate the role of ANP32E, which is shown to only play a minor role in supporting IBV polymerase activity. Finally, binding of polymerase and ANP32 proteins is investigated.

The work is well-structured, follows a clear line of thought and is presented in an understandable way, although language would benefit from some enhancements. The conclusions are valid and the data are of some interest to the community. However, much of the work is rather confirmatory with incremental advance in our understanding of the role of ANP proteins in influenza B virus infection at this stage of analysis. This is in part also due to the strong focus of the work on the use of ectopically expressed genes and less on the situation in virus-infected cells.

Response:

We thank the reviewer for reading our manuscript and giving the comments. But we do not agree with them. The reasons were also listed in the previous submitted appeal letter.

We cannot agree and donot understand the logic. What is the reviewer’s current understanding of the role of ANP proteins in influenza B infection and its interspecies restriction? Please show some evidence if possible. Why the use of ectopically expressed genes is the reason of short of incremental advance? We read comments very carefully and we donot agree with the reviewer’s logic. Did the reviewer think that the assays we used are not enough to make conclusion (but the reviewer already agree the conclusions are valid)? Most experiments are based on single or multiple gene knockout cell lines, by which we evaluated the endogenous and exogenous proteins’ function. We also incorporate other methods such as co-IP and SPR to investigate the detailed interaction between ANP32 proteins and viral polymerase, which were not mentioned by the reviewer. More importantly, our major finding is the roles of ANP32 proteins from different species in supporting IBV polymerase and the molecular basis of interspecies restriction. We are not convinced with these comments which ignore the major findings and seem “fake logic”.

1.)	A major result of this manuscript, the dependence of influenza B virus propagation on ANP32A and -B proteins, has already been demonstrated (e.g. Staller et al., J.Virol. 2019). Due to its depth of analysis, the current manuscript extends this finding, but is rather confirmatory in character. The second novel aspect is the analysis of the role of ANP32E. As the authors show and state themselves, however, the contribution of this isoform to polymerase function is smaller compared to the previously identified major host range determinants ANP32A and B.

Response:

The reviewer said the main finding of our manuscript has been demonstrated by Staller et al., J.Virol. 2019. We don’t agree with this point. I have attached the Staller’s paper (J.Virol. 2019) in which very limited data present in Figure 1F (This figure showed human ANP32A and ANP32B had impact on influenza B polymerase activity. while the poor expressing level of viral NP in this experiment makes the result doubtful.), figure 2D (showed overexpress of human ANP32A and human ANP32B support influenza B polymerase activity), and figure 6C (showed Murine ANP32A can support IBV polymerase). These were only three standard polymerase activity assays without other evidence. These data only showed the human ANP32A and B, and murine ANP32A might support an IBV polymerase activity. Importantly, there are lots of unknown questions on the interaction between ANP32 proteins and IBV replication, especially the contribution of the other member of ANP32 protein like ANP32E, the specie specific roles of ANP32 proteins in supporting IBV replication and their molecular basis. Our study focused on these questions and give answers of these questions which improves the understanding of ANP32 proteins in supporting IBV replication and host determination. (as commented by reviewer 1 and reviewer 2). We are really disappointed that the reviwer3 ignored the major results and did not make judgment seriously.

The reviewer said “The second novel aspect is the analysis of the role of ANP32E. As the authors show and state themselves, however, the contribution of this isoform to polymerase function is smaller compared to the previously identified major host range determinants ANP32A and B.” It is true that ANP32E has limited ability to support IBV and no activity to IAV. This is a new finding point in the field and we further identified “ANP32E functions poorly due to variation at aa129, and the species-specific absence of an acidic stretch further eliminates activity in birds, suggesting ANP32E is a major factor restricting IBV host range”( as the reviewer 2’s comments). The reviewer 3 ignored these finding and seems has lots of misunderstanding, or maybe not familiar with this field.

2.)	The major body of evidence gained in this manuscript relies on standard polymerase activity assays. These are performed with skill and the immunoblots showing control expressions are of high quality. However on top of that, only one panel (Fig.1D) investigates the impact of ANP32 proteins on virus replication. Also, little significant novel insight is gained here, since deletion of ANP32E in cells already lacking ANP32A and B does not have an impact on virus replication. The interaction analyses in figures 7 and 8 again only marginally increase knowledge.

Response:

We used standard polymerase activity assay and protein-protein interaction analysis by co-IP, western blotting and SPR assay to demonstrate the interaction between ANP32 and viral polymerase. The figure 1D confirmed the knockout of the ANP32 proteins impact on viral replication. Is the reviewer saying this is not enough to make the conclusion? We believe the reviewer misread the data. Because of the TKO created, we can evaluate the contribution of each protein in supporting viral replication, this data is very important to illustrate the functions of ANP32 family proteins in IBV replication. Figures 7 and 8 provide novel insights into critical amino acids of ANP32A and ANP32B in supporting IBV replication, and the potential dynamic difference between chicken ANP32A and human ANP32A in interacting with IBV polymerase. This finding for the first time shows that the binding affinity is related to the ability of ANP32 proteins in supporting IBV replication.

--------------------

<b>Part II – Major Issues: Key Experiments Required for Acceptance</b></br>Please use this section to detail the key new experiments or modifications of existing experiments that should be <u>absolutely</u> required to validate study conclusions.<br/><br/>Generally, there should be no more than 3 such required experiments or major modifications for a "Major Revision" recommendation. If more than 3 experiments are necessary to validate the study conclusions, then you are encouraged to recommend "Reject".<br/><br/>

Reviewer #1: No major issues identified

Reviewer #2: 1.	Experiments studying interactions between IBV polymerase and ANP32s are underdeveloped and the claims need to be softened

Response:

We thank the reviewer for the suggestion. We are sorry for our blunt statement. We have reworded the sentences (line 343-347).

a.	The authors use results from SPR to calculate Kd and claim avian ANP32A binds IBV polymerase ~2.5 fold less well than human ANP32A. However, these data have no statistics associated with Kd calculations. The curve in Fig 8F does not correlate with the individual data in 8A-C. If anything, data in 8C suggest higher response rates for huANP32A+33 than for ANP32A.

Response:

Thanks for the comments. We apologize for the mistake made to Fig. 8A and 8C which were mislabeled. We have confirmed the related data and corrected the figures.

b.	Minor differences in Kd determined by SPR can be caused by differences in protein quality or concentration. Data showing the purity and equivalent concentration of ANP32s is needed. It would also be useful to understand how well IBV polymerase proteins are expressed, as the methods say these proteins are captured on the chip directly from cell lysate.

Response:

Thanks for the suggestions. The ANP32 proteins were fusion expressed at downstream of GST-HRV3C peptide in a pCAGGS vector in TKO cells and purified using Glutathione Sepharose 4B and then digested by PreScission Protease (Beyotime). Purified ANP32 proteins were diluted to 100ug/ml and 1ug of each purified protein was checked by SDS-PAGE and western blotting. All ANP32s were showed well expressed and with good purity (S5 Fig A). IBV polymerase PB1, PB2 and PA-His were expressed in 293T cells and followed by purification with Ni Sepharose (GE). The expression and components of IBV polymerase proteins were identified by SDS-PAGE and mass spectrometry (S5 Fig B).

c.	While a major new claim is that ANP32E support IBV polymerases, ANP32E was not included in any of the binding assays.

Response:

Thanks for the comments. We have compared the binding abilities to IBV polymerase of ANP32Es with that of ANP32A and ANP32B. We have added the description in line 310-316 and Fig. 7E. We found that huANP32E and chANP32E showed weak binding to IBV polymerase compared with huANP32A/B. E129N mutation of huANP32E and chANP32E increased their binding abilities to the same level of huANP32A/B, while the 10 amino acids difference between huANP32E and chANP32E does not influence the binding. These results are consistent with their supporting abilities to IBV polymerase.

d.	IBV polymerase interacts by coIP with both huANP32A and chANP32A, yet only huANP32A supports function. If the authors are able to confirm minor differences in binding proposed in Fig 8, would these even be relevant to IBV pol function in cells?

Response:

Thanks for the question. We observed that IBV polymerase strongly interacts by coIP with both huANP32A and chANP32A at similar level (Fig. 7D and 7E). We are not able to find mirror differences by co-IP. While the SPR assay showed huANP32A has stronger binding dynamics than that of chANP32A, which could be relevant to IBV polymerase function and need further evaluations.

e.	Given the limited support of Fig 8, lines 388-399 in the Discussion are too speculative. In addition, SPR was done in the absence of viral RNAs, thus different functional states of the polymerase cannot be assumed.

Response:

We thank reviewer for the correction. We have reworded the related sentences.

2.	The authors identify a 10aa insertion of acidic residues present in mammalian ANP32E that they nicely demonstrate is important for the low levels of huANP32E activity. While murine ANP32E is tested in cells, it is conspicuously absent from their alignment.

Response:

Thanks for the question. We have added the murine ANP32E in the alignment.

a. Alignments show that muANP32E lacks this insert. This should be addressed in the text.

Response:

muANP32E lacks the 10aa insertion, which is different from other mammalian ANP32E. muANP32E has a double aspartic acid insertion at position 162-163, which is absent in other mammalian and avian ANP32E. muANP32E showed mild support to IBV polymerase compared with the ANP32Es from other species and the reason should be further studied. We have addressed this question in the updated version (line 261-266).

b. Fig 4A,B; it is not clear why muANP32B is used as a control here. Staller et al. 2019 showed that muANP32B actually encode 129S 130D, which is different than the more common 129N 130D found in huANP32B, huANP32A, and chANP32A. Fig 6 here goes on to show that the identify of aa129 is important for ANP32E function. This difference in muANP32E should also be addressed in the text.

Response:

Thanks for the comments. Murine ANP32B encodes 129S 130D, which are different from other mammalian ANP32A or B (129N 130D). The Asparagine (N) and Serine (S) are all polar uncharged amino acids and both signature showed good support to IBV polymerase, while chANP32B has 129I 130N signature and showed less support to IBV polymerase. Therefore we compared the 129-130 sites between ANP32A and ANP32E proteins and identified that all the ANP32E proteins from different species including huANP32E, chANP32E, and muANP32E have 129E signature, which limited the ability in supporting IBV polymerase. We have added the description in the text (line 209-213, line 275-278)

c. care should also be taken to make clear that the 10aa insertion confers species-specific function, while residue 129 is the dominant change controlling activity regardless of the host.

Response:

We thank the reviewer for the comments. We agree and have added the discussion in the text (line 389-397)

3.	The authors claim that ANP32s are a major barrier preventing IBV replication in birds. However, this was never tested. Does expression of huANP32A or B (or perhaps chANP32E_E129N) allow IBV replication in birds, or do other barriers still exist?

Response:

We thank the reviewer for the comments. We are sorry for the inaccurate language. We have revised the claim. We did infection experiments in DF1 cells with or without expression of huANP32B using an IBV strain and found that the expression of huANP32B in DF1 cells could enhance IBV replication. We have added this result as Fig 2C. Although our results showed different support ability between human and chicken ANP32 proteins, we could not exclude other barriers existed in bird to limit IBV infection.

Reviewer #3: (No Response)

--------------------

<b>Part III – Minor Issues: Editorial and Data Presentation Modifications</b></br>Please use this section for editorial suggestions as well as relatively minor modifications of existing data that would enhance clarity.<br/><br/>

Reviewer #1: Lines 163-167. Please check this statement. I thought that mammalian ANP32s were ‘short’, while avian ones were ‘long’? Statement seems contrary to line 160.

Response:

Thanks and we apologize for the mistake. We have corrected the sentence in the revised manuscript.

Line 185. Please clarify what is meant by chickens have an ANP32A isoform that lacks the 4 hydrophobic amino-acids – are the authors referring to multiple isoforms of ANP32A? Please make this clearer – perhaps discuss different isoforms earlier in the introduction to make it obvious to the reader that they exist. Also, chickens are reported to have a short isoform lacking the entire ‘insertion’ – eg like del33. Please comment.

Response:

We thank the reviewer for the comments. We have added description of multiple isoforms of ANP32A in the introduction section (line 80-85) and added discussion in line 414-425.

Line 188 – please specific what ‘alter’ means – enhance, reduce? Not clear what meant here.

Response:

Thanks and we have change the “alter its support to IAV polymerase” to “reduce its support to avian IAV polymerase”

Line 193 – other than chickens, some birds have higher ratios of the ‘short’ ANP32A isoform to the ‘long’ isoform (Baker et al, Domingues et al) – can the authors make a definitive statement whether these birds could support IBV replication? In the discussion, perhaps comment on the amino-acid compositions in ANP32B and ANP32E of the bird species that are dominant in expressing ANP32Adel33 isoforms. Would their ANP32B/E molecules also suggest a support for IBV polymerase?

Response:

Thanks for the comments. The ratios of the ANP32A isoforms in different avian species are vary. Chicken is reported to have higher ratios of ‘long’ isoforms, while some birds have higher ratios of ‘short’ form. The function and contributions of different ANP32A isoforms to support IBV replication in different birds need to be further studied. The virus replication in a host depends on many factors and we could not make a definitive statement based on the current understanding. The chicken ANP32B and ANP32E are all in ‘short’ form and they both have limited abilities to support IBV polymerase, because the chicken ANP32B has 129I 130N, and chicken ANP32E has 129E, which limited their support to IBV polymerase.

Figure 4 – what is muANP32B? Murine? Why is its apparent size larger than huANP32B? Doesn't seem to be referred to anywhere in the text of Figure legend.

Response:

Sorry for the confusion and the missing information. The muANP32B is murine ANP32B. It is larger than huANP32B because of an extra-long C terminal as showed below. We have added text in line 209-213 and in the figure legend.

Reviewer #2: 1.	Fig legends 3, 4, 5B,C, do not indicate which cell lines are being used. This reviewer assumes the experiments are in TKO. If not, I will have to reconsider my interpretation of the data

Response:

We are sorry for this missing information and the TKO cells were used in these experiments. We have updated the figure legends.

2.	A model or table in the Discussion indicating the functionality of avian and human ANP32s with huIAV, chIAV or huIBV would be a useful addition to help solidify current and previous findings.

Response:

We thank the reviewer for this suggestion. We have added a model figure (Fig 9) and discussion to summarize the interaction between ANP32 proteins and different IAV and IBV polymerase.

3.	Table 1 was not present in the manuscript received for revue. However, given the concerns about SPR above, it is not clear how much Table 1 contributes.

Response:

We apologize for missing of the table 1 in the text. Table 1 showed values of dissociation rate constants (kd) and the association rate constants (ka). We would update this table as the supplemental table 1.

4.	Please indicate that Staller et al. 2019 showed that ANP32B enhances influenza B polymerase activity.

Response:

Thanks for the comments. We updated the relevant text in line 211-213.

5.	Statistics throughout. Reporting SEM for technical replicates is not accurate, please use SD to illustrate error bars and to run statistical tests on. Number of replicates are not indicated.

Response:

Thanks and we have rerun the statistical tests and updated the figures with SD.

6.	Fig 3 misrepresents where the duplication event occurred – chANP32A duplication was downstream of SIM sequence, so “repeat 1” should have matching alignment of ch and hu. See Long 2015 Extended Data Fig 8 or Domingues 2017 Fig 2. Fig S3 alignment is inaccurate for the same reason. Figure S3 Legend indicates numbering with respect to huANP32A but this is not how the alignment is presented.

Response:

Thanks and we have revised the figures.

7.	ANP32B and ANP32E were independently shown to be essential (in 1 of 5-7 screens each; <http://ogee.medgenius.info/browse/).> Please discuss the health of TKO cells.

Response:

Thanks for the comments. Although the ANP32B and ANP32E were independently shown to be essential, we have tested the cell viability of TKO and the cell was in good health. We added this data as S1 Fig.

8.	Line 292 – these results are not “suprising” as they have already been reported for IAV:ANP3A interactions by others.

Response:

Thanks and we have deleted the word.

Reviewer #3: (No Response)
