# Supplementary material for: Selective usage of ANP32 proteins by influenza B virus polymerase: Implications in determination of host range
Source: PLoS Pathog. 2020 Oct 12;16(10):e1008989. doi: 10.1371/journal.ppat.1008989 (PMC7580981; doi:10.1371/journal.ppat.1008989)
Supplement: Attachment — Submitted filename: Respone to the reviewers 20200912.docx [file ppat.1008989.s007.docx]

Reviewer Comments (if any, and for reference):

Reviewer's Responses to Questions

**Part I - Summary**
Please use this section to discuss strengths/weaknesses of study, novelty/significance, general execution and scholarship.

Reviewer #1: I am still very positive about this manuscript, and believe that it adds significantly to the field and our understanding of species-specific functional interactions between ANP32 proteins and influenza viruses. The data are novel, experiments well-executed, and the manuscript provides new detailed insights into the molecular basis of ANP32E usage, as well as avian versus mammalian ANP32A/B interplay with the polymerases of both influenza A and influenza B viruses. The findings have clear implications for trying to understand host restriction. Importantly, all of my previous comments, which were very minor, have been addressed satisfactorily.

Reviewer #2: The authors have addressed my major concerns. I appreciate their attention to each of the points raised and thorough responses.

Reviewer #4: The results presented in this manuscript advance our understanding of the important role of ANP32 proteins in the regulation and adaptation of the polymerase activity of Influenza A but most importantly on Influenza B viruses. It provides compelling and solid evidence, that ANP32 proteins promote IBV polymerase activity in human cells in a protein specific manner, which is in some parts divergent from the effects seen on the IAV polymerase.
Many of the primary research questions in this manuscript have been addressed before by Staller et al. 2019, Journal of Virology using similar methodology. Thus the originality and novelty of the subject is limited. In contrast to Staller et al. who reporter a certain degree of functional diversity between ANP32A and B proteins on the IBV Polymerase activity, the results in this manuscript show functional redundancy of both proteins in human cells. These differences may be attributed to the choice of IBV strain, cell type and method of generating KO clones and are in general interesting but not ground breaking.
The investigation of ANP32E, which shows limited support for the IBV polymerase is new and interesting, however, compared to ANP32A/B it appears to be of minor importance and its role in the species restriction of IBV to humans remains uncertain. The chosen methods mostly assess protein functions under non-natural (overexpression, purified) conditions and only very few assays verify results in the context of a viral infection, which overall is a weak point.

In the light of these shortcomings I would suggest submission of this manuscript to a more specialized journal.

In addition, I would like to pronounce my concerns about the response of the authors to reviewer #3, which is in parts rude and inappropriate. In a peer review process authors and reviewers should adopt a professional and supportive attitude even if the outcome is not as preferred.
To avoid any misinterpretation: My evaluation of the presented scientific work is by no means affected by this statement.

Response:

We thank the reviewer for the comments. We agree that in a peer review process authors and reviewers should adopt a professional and supportive attitude. We realized that the language usages might be improper in some part of the appeal letter regarding the basis of judgment made by the reviewer 3. We had no intention of offending the reviewer. I apologize if the reviewer feels uncomfortable.

**Part II – Major Issues: Key Experiments Required for Acceptance**
Please use this section to detail the key new experiments or modifications of existing experiments that should be absolutely required to validate study conclusions.

Generally, there should be no more than 3 such required experiments or major modifications for a "Major Revision" recommendation. If more than 3 experiments are necessary to validate the study conclusions, then you are encouraged to recommend "Reject".

Reviewer #1: None

Reviewer #2: (No Response)

Reviewer #4: (No Response)

**Part III – Minor Issues: Editorial and Data Presentation Modifications**
Please use this section for editorial suggestions as well as relatively minor modifications of existing data that would enhance clarity.

Reviewer #1: None

Reviewer #2: (No Response)

Reviewer #4: General remarks:
1. What is the rational of using a H7N9 IAV derived polymerase? This subtype has only recently jumped to humans but is not stably adapted (not circulating) to the human population in comparison to the H1N1 and H3N2 subtypes. Please include some information on why this strain was chosen as a comparison to IBV, which is a seasonal strain and much more stable adapted to humans.
Response:

We thank the reviewer for the comments. The H7N9 virus is a typical influenza A virus which can transmit from avian species to humans in some cases. The polymerase of H7N9 chosen has human adapted mutation (PB2 E627K) and has similar activity and dependence on ANP32 proteins compared with H1N1 and H3N2 seasonal viruses, which have been demonstrated in our previous paper (Zhang H, et. al. 2019 JVI, doi: 10.1128/JVI.00174-19.). We have included a description in the revised text (Line 117-119).

2.The labeling of the y-axis in all figures displaying IAV polymerase activity using the polymerase reconstitution assay is wrong and needs to be changed to: Firefly Luciferase activity instead of Firefly Polymerase.
Response:

We thank the reviewer for the correction. We have corrected the labeling of the y-axis in all figures in the revised manuscript.

Specific remarks:
1. lines 76-78: What means “the ANP32 family MAINLY includes ANP32A, ANP32B and ANP32E.”? Does this refer to in most species? Or expression levels? Are there other ANP32 proteins present? Please specify this statement.
Response:

We thank the reviewer for the questions and we have change the sentence to “The ANP32 family includes three conserved family members: ANP32A, ANP32B, and ANP32E.”

2. lines 78: This sentence is misleading. Please acknowledge that also other groups have contributed to this finding.
Response:

Thanks for the comments. We have reworded the sentence and updated the references in line 78.

3. Figure 1: Please indicate the subtype of the FluA polymerase as this is a relevant information for this assay.
Response:

Thanks, and we have added the subtype in revised Figure 1.

4. Lane 123: what means: the last “functional member”? What function does this refer to? Please describe this in more detail. Also lane 124: what means “considered to be an important member of the ANP32 family”? Are there other non-important members? What is this hypothesis based on? Please provide more details.
Response:

We are sorry for the confusion. We have corrected the sentences as “We speculated therefore that the conserved ANP32 family member, ANP32E, may contribute to the support of IBV polymerase activity. ANP32E has a similar sequence to those of both ANP32A and ANP32B, but is not able to support IAV replication.” in the revised manuscript.

5. Line 149: what means ANP32As? Is that s a typo? Please correct.
Response:

Thanks and we have corrected the word in the revised manuscript.

6. Figure 2: The title of this figure is misleading as it states that only ANP32As from different animals are investigated which is not true
Response:

We apologize for the mistake. We have corrected the title in the revised manuscript.

7. Figure 2B: why is there no ANP32 protein band in the empty vector control lane? These are wild type cells and should express detectable levels of endogenous chicken ANP32 proteins. Please explain and interpret this result.
Response:

We thank the reviewer for the comments. We use anti-Flag tag antibody to test overexpression ANP32 proteins, so the endogenous chicken ANP32 proteins can not be detected. We have updated the figure legends.

8. Figure 2C: Why were virus growth curves performed at 37°C? For IBV 33°C is commonly used as it also support much higher titers. I wonder whether overexpression of human ANP32B also has a positive effect at 33°C? Can you provide data on this?
Response:

We thank the reviewer for the comments. Although normally the IBV was grew in MDCK in 33°C, we actually compared the growth curves of IBV uses in this research in DF1 cells and 293T cells at both 37°C and 33°C, and we did not observed statistic difference between the two temperature conditions. The reason could be different cell or virus isolate used. We have updated the figure 2C with these results.

9. lane 174: Please correct the sentence to …”that ARE all in short form…” and remove the are in lane 176.
Response:

Thanks and we apologize for the mistakes. We have corrected the sentence in the revised manuscript.

10. Are there any other aa mutations between the different mammalian and avian type ANP32A proteins beside the 33 aa insertion? Please state so clearly in the primary text (in the respective section) as these mutations can also affect the protein function on the polymerase.
Response:

We thank the reviewer for the comments. There are some substitutions between the different mammalian and avian type ANP32 proteins beside the aa insertion. There is also an ANP32A isoform with a 29 aa insertion in most avian species that is different from mammalian species. The avian ANP32B harbors unique amino acids at 129/130 site which aborted its supporting activity to IAV polymerase (descripted in line 211-214). The swine ANP32A protein has some special mutations can affect the protein function on avian viral polymerase. We update the description in line 80-82.

11. Which cells were used in figure 3d? Please also include this information in the text and legend.
Response:

We thank the reviewer for the comments. We used TKO cells in the experiments. We have updated this information in the text and the figure legend.

12. lane 187: correct IVB to IBV
Response:

Thanks and we apologize for the mistake. We have corrected the word in the revised manuscript.

13. lane 204: I understand that chicken ANP32B also lacks the 33 aa insert. Is that correct? But there is a size shift in the westernblot bands compared to human ANP32B indicating addition amino acids. Please add this information to the beginning of this paragraph.
Response:

We thank the reviewer for the comments. The chicken ANP32B (262 aa in length) has a longer (11 aa) at C terminal of LCAR region than that of huANP32B (251 aa in length). We added this information in the revised manuscript.

14. line 306: please change mutant to mutations
Response:

Thanks and we have corrected the word in the revised manuscript.

15. figure 7D: What happens if the LCAR region of ANP32A is removed? Is binding to IBV polymerase then abolished? Is that a common mechanism for all ANP32 proteins? Why was the ANP32A_165T mutant not tested in the co-IP assay but in the SPR?
Response:

We thank the reviewer for the comments. We had the co-IP assay data showed that, similar to ANP32B, if the LCAR region of ANP32A is removed, the binding to IBV polymerase is abolished. So we used ANP32A_165T in the SPR because we think it is a good control for human and chicken ANP32A proteins.

16. line 360: I assume that the authors meant write morbidity instead of mobility? Please correct.
Response:

Thanks and we apologize for the mistake. We have corrected the word in the revised manuscript.

17: line 362: “polymerase replication” is a confusing description. Please substitute with “polymerase activity” Please substitute
Response:

Sorry for the confusion and we have corrected the sentence in the revised manuscript.

18. overall the grammar, spelling and wording in the discussion part is of lower quality than the previous parts of the manuscript and needs major improvement (e.g. lines 417-425)
Response:

We thank the reviewer for the comments and we have corrected the sentence in the revised manuscript.

19. lines 420-425: The authors claim that the amount of mammalian-like ANP32A isoform in DF1 cells is not enough to support IBV replication. But in Figure 2C a growth kinetic of B/Yamagata/PJ/2018 is presented. So in principle, IBV replication seems to be supported in theses cells. Please rephrase this part of the discussion accordingly.
Response:

We thank the reviewer for the comments and we have reworded the sentences.

20. line 427: “chicken IAV”? does this mean avian IAV strains or do you really mean IAV from chicken? Please be precise.

Response:

Thanks and we apologize for the confusion. It means avian IAV strains and we have corrected the sentence in the revised manuscript.
